# Scoping review of the association between bacterial vaginosis and emotional, sexual and social health

**DOI:** 10.1186/s12905-023-02260-z

**Published:** 2023-04-07

**Authors:** Judith Brusselmans, An De Sutter, Brecht Devleesschauwer, Hans Verstraelen, Piet Cools

**Affiliations:** 1 Department of Diagnostic Sciences, Faculty of Medicine and Health Sciences, Ghent University, Corneel Heymanslaan 10, Ghent, 9000 Belgium; 2 Department of Public Health and Primary Care, Faculty of Medicine and Health Sciences, Ghent University, Corneel Heymanslaan 10, Ghent, 9000 Belgium; 3 Department of Translational Physiology, Infectiology and Public Health, Ghent University, Merelbeke, Belgium; 4 Department of Human Structure and Repair, Faculty of Medicine and Health Sciences, Ghent University, Corneel Heymanslaan 10, 9000, Ghent, Belgium; 5 Department of Epidemiology and Public Health, Sciensano, Brussels, Belgium

**Keywords:** Bacterial vaginosis, Body image, Douching, Self-esteem, Stress, Qualitative methods, Quality of life, Vaginitis, Sexual life, Social life, Scoping review.

## Abstract

**Background:**

Bacterial vaginosis (BV) is a condition that, if symptomatic, is characterized by discharge and odor, with high recurrence rates even when treated. This study aims to review what literature exists on the association between BV and the emotional, sexual, and social health of women.

**Methods:**

MEDLINE, Embase and Web of Science databases were searched from inception until November 2020. Studies reporting an association between women’s emotional, sexual and/or social health and symptomatic BV in a qualitative and/or quantitative manner were included. Selected studies were divided in three categories, i.e. reporting on the emotional, sexual and/or social association. All studies were critically evaluated and discussed.

**Results:**

Sixteen studies were included. Concerning emotional health, we found eight studies that calculated the association between stress and BV, in four this was statistically significant. Four qualitative studies on emotional health showed that the severity of the symptoms influenced the impact on women’s lives. All studies on sexual health reported that many women experienced an impact on their relationship and sexual intimacy. Results for social life ranged from no association found to most of the study population showing avoidance behavior.

**Conclusion:**

This review shows that symptomatic BV can be associated with diminished emotional, sexual, and social health, but there is too little evidence to state the extent of this association.

**Supplementary Information:**

The online version contains supplementary material available at 10.1186/s12905-023-02260-z.

## Background

Bacterial vaginosis (BV) is a gynecological condition that has been described as a dysbiosis of the vaginal microbiome, an infection or an inflammatory disease [1]. In women with BV, the protective lactic-acid producing lactobacilli of the vaginal microbiome are largely replaced with anaerobes such as *Gardnerella vaginalis* and *Atopobium vaginae*, [2] leading to symptoms of malodor and discharge [3]. It is estimated that BV affects 23 to 29% of women in the general population worldwide, [4] but in groups at high risk for sexually transmitted infections (STI), the prevalence can be as high as 70% [5]. In up to 83% of women, BV is present without symptoms [6]. When symptoms are present, the malodor is often described as fishy [7] and the milk-like discharge as thin, homogenous [8] and profuse [7]. These symptoms may potentially affect a positive body image, which is important for high self-esteem and healthy sexuality [9]. The management of BV relies primarily on the eradication of the pathogenic bacteria with selected antibiotics (metronidazole or clindamycin), [10] with cure rates of 80–90% in the first month after therapy, but after 12 months recurrence rates approach 60% and higher [11]. The high recurrence rates and lack of long-term treatment strategies can be frustrating to both patients and care providers.

The aim of current study was to review what literature exists on the association between BV and the emotional, sexual, and social health of women. Analysis of this information can be of importance to obtain a comprehensive picture of the overall disease burden of BV. It can also help explain why self-help remedies such as douching, which are more likely to worsen the condition than to help improve it, are often continuously used.

## Methods

### Search strategy

The Preferred Reporting Items for Systematic Reviews and Meta-analyses extension for Scoping Reviews (PRISMA-ScR) guidelines [12] were followed in the reporting process of this scoping review, using an a priori defined study protocol that can be accessed on request. Studies were searched in the MEDLINE (through PubMed), Web of Science Core Collection and Embase (through embase.com) databases. The search terms used were ‘bacterial vaginosis’ in combination with ‘burden’, ‘discomfort’, ‘impact’, ‘perceived stress scale’, ‘psychology’, ‘quality of life’, ‘self-esteem’, ‘sexual life’, ‘shame’, ‘social life’ or ‘stress’. The bibliographic reference list of included studies was also hand searched for relevant papers. The search results were downloaded into an Endnote database.

### Study selection

After removing all duplicates, studies were screened by one reviewer (JB) using the information in title and abstract only to identify studies for full text evaluation. Studies were included if they reported an association between emotional, social and/or sexual health and BV in a qualitative and/or quantitative manner. Papers in English, French, Spanish, German and Dutch were considered. Papers were included if published before the 14th of November 2020. Randomized controlled trials, cohort studies, case-control studies and qualitative studies were included. Reviews, conference abstracts, comments, guidelines, case reports, theses, dissertations, and case series were excluded. Studies that reported on the effect of vulvovaginal symptoms in general, but that did not present separate data on BV and did not allow the review team to calculate so, were not included. The reason for exclusion of articles was recorded and categorized. In case of doubt, advice was asked from the research team.

### Data charting and analysis

According to definitions put forward by Feller and colleagues (emotional health), [13] World Health Organization (sexual health) [14] and Russel and colleagues (social health), [15] papers were classified as follows:

- under emotional health studies that reported on emotions and feelings, stress, ideas, concerns and expectations and/or quality of life in general;
- under sexual health studies that involved sex life, sexual self-esteem and/or romantic relationships;
- under social health studies on interaction with surroundings, everyday life, hobbies, friends and/or job and colleagues.

From each study, the following information was charted: first author, year of publication, country where the study was carried out, study design, qualitative or quantitative study, study period, study population, total number of study participants, age (range), definition of BV, prevalence of (recurrent) BV, physical impact (prevalence of symptoms in general, prevalence of odor, prevalence of discharge, prevalence of other symptoms, duration of symptoms), prevalence of side effects of medication, prevalence of sexual impact, prevalence of emotional impact, definition of stress, prevalence of stress, prevalence of social impact, prevalence of frequent use of hygienic practices, association stress-BV, qualitative data on the emotional, sexual, and/or social health of women, and whether confounders were accounted for, and if so, which confounders and how these were handled. The charted data was summarized and discussed in the respective categories of emotional health, sexual health and social health.

## Results

**Fig. 1:**
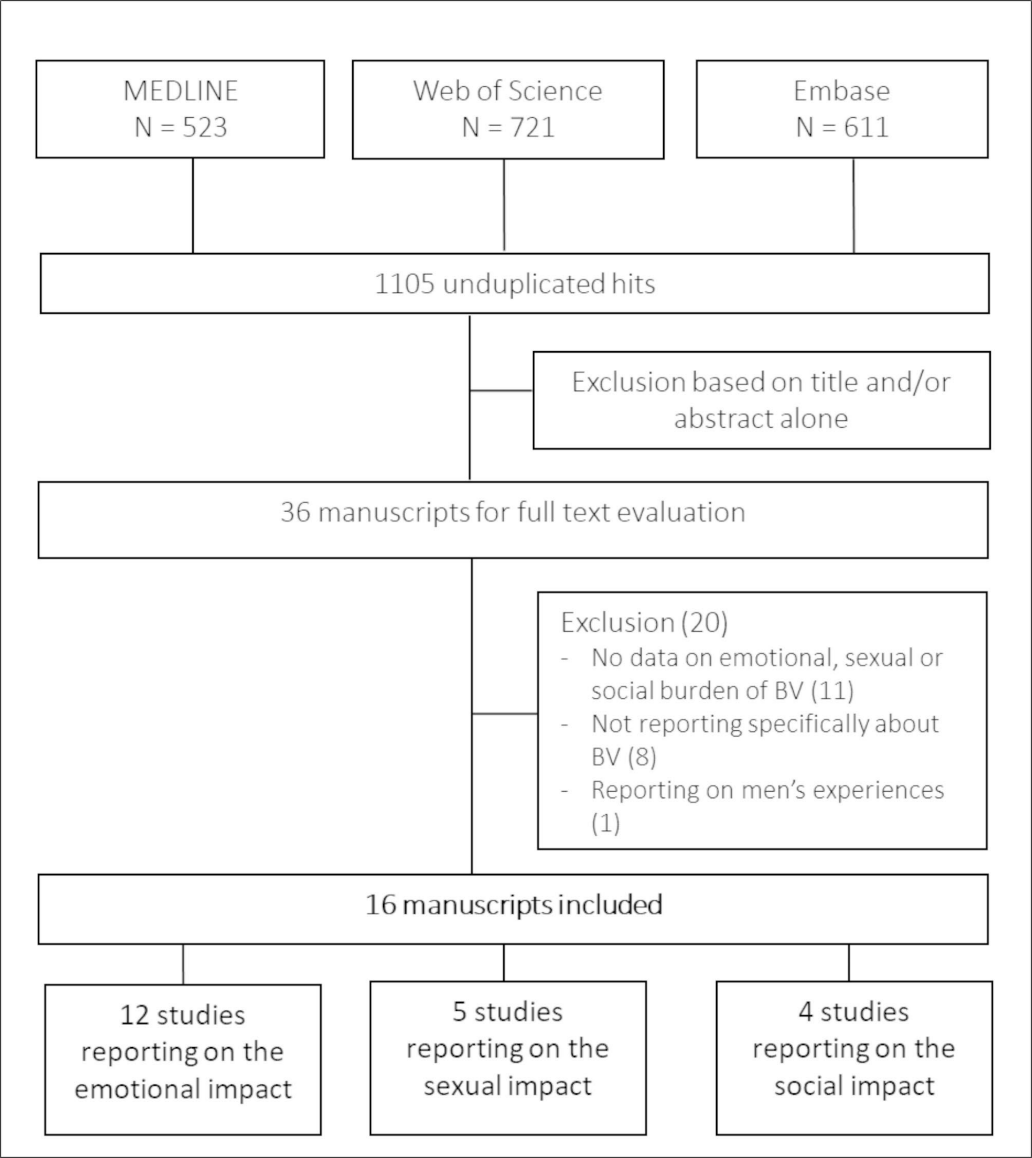
Flowchart of the study selection

We obtained a total of 1105 unique citations by searching the MEDLINE, Embase and Web of Science Core Collection databases. A total of 1069 studies were excluded based on title and/or abstract alone, leaving 36 studies for full text evaluation (Fig. 1; Additional file 1). Of these, eight were studies about vulvovaginal diseases, but did not present separate data for BV. Eleven studies did not include any information about sexual, social, or emotional health in relation to BV. One study involved the experiences of the male partners of women with BV. Sixteen studies were included, [7, 11, 16–29] of which twelve studies regarding emotional health, [7, 16–22, 24, 25, 27–29] five on sexual health [7, 11, 16, 18, 23, 27], and four involving social health [7, 11, 16, 26, 27]. Nine out of sixteen manuscripts reported on studies that were conducted in the US, four were conducted in Australia, the three remaining studies were carried out in India, Kenya, and the UK. The studies included were very heterogeneous in terms of study population, study goal and methodology.

### Emotional health

Of the twelve studies we included in the category ‘emotional health’, eight studies specifically addressed stress as a determinant of BV (Additional file 6). Four of these studies found a statistically significant association between stress levels and BV [20, 24, 25, 29]. Nelson and colleagues assessed the level of stress separately in women with symptomatic and asymptomatic BV [25] and reported significantly lower mean stress levels in women with asymptomatic BV compared to symptomatic BV. Because of the cross-sectional nature of these studies [19–22, 25, 28, 29] except one, [24] no meaningful inferences can be made about the direction of these associations.

Another four studies involving emotional health were reported in five manuscripts that obtained data on emotional health and BV (Table 1) [7, 16–18, 27]. Two studies specifically included women suffering from recurrent episodes of BV [7, 17, 27]. Two studies used questionnaires to obtain data, [18, 27] of which one also conducted interviews [27]. Two studies only reported qualitative data out of interviews and did not report the number of women who suffered an impact [7, 16, 17].

**Table 1:** Summary of studies reporting on the emotional impact of BV.

Study population	Sample size	Definition of (recurrent) BV	Study design	Major findings	Study ID
Women with recurrent BV	35	≥ 2 self-reported episodes per year	Qualitative	Over 2/3rd of women experienced moderate to severe impact ^a^.	Bilardi et al. 2013 [7]
Women with recurrent BV	35	≥ 2 self-reported episodes per year	Qualitative	The majority of women reported frustration and dissatisfaction with current treatment and clinical management ^a^.	Bilardi et al. 2016 [17]
NP, symptomatic BV	33	Clinical diagnosis based on patient-reported odorised discharge	Qualitative	Emotional and physical despair was reported by every woman in this study.	Anstey Watkins et al. 2019 [16]
African-American women with recurrent BV	20	≥ 2 proven episodes (lifetime) using Amsel criteria	Qualitative/ quantitative	In interviews, women expressed feeling acutely stressed and experienced increased sensitivity and depression during episodes of BV ^a^.	Payne et al. 2010 [27]
NP, symptomatic BV	404	Nugent score 3–10 and ≥ 3 Amsel criteria	Quantitative	16.5% and 75.9% of women were respectively somewhat concerned to concerned/very distressed because of the effect of their symptoms on their quality of life.	Bradshaw et al. 2013 [18]

**Legend.** NP, non-pregnant. ^a^ Author’s data did not allow us to present specific numbers here

In all four studies, most of women experienced a moderate or severe emotional impact of BV on their lives. Women expressed feeling acutely stressed and experiencing increased sensitivity and depression when having an episode of BV [16, 27] and feeling relieved when they did not [7]. Most women felt embarrassed, self-conscious, and uncomfortable [7, 16, 27]. Many expressed feelings of shame and disgust, adversely impacting their self-esteem and confidence [7, 16]. They suffered from the societal stigma around sexuality and STIs. For some women, having recurrent BV led to worrying thoughts about long-term (reproductive) sequelae [7]. Women who experienced BV, especially recurrent episodes of BV, generally felt frustrated and confused [7, 16, 17, 27]. The interviews of the qualitative studies showed different reasons for this frustration and confusion. First, although women tried different treatments and (expensive) self-help remedies, most of them did not perceive any amelioration of the symptoms and frequency of recurrences [7, 16, 17]. Second, women did not know what triggered their symptoms and felt having no control over it. Some women believed they would always have BV and there was no remedy for it [7, 16, 17]. Third, a lot of women just wanted answers for the questions they had regarding BV, however they felt that clinicians themselves had little knowledge on the causes and treatment options [17]. This inadequacy and inconsistency of clinical information built to the frustration and distress of these women [17]. Fourth, women were frustrated because the psychosocial impact of BV was often not recognized by people around them [17].

### Sexual health

Five studies reported on sexual health and BV [7, 11, 18, 23, 27] and are summarized in Table 2. Two studies specifically included women suffering from recurrent episodes of BV [7, 27]. Four studies used questionnaires regarding symptoms, practices, and impact [11, 18, 23, 27]. Two studies collected qualitative data by interviewing women [7, 27].

**Table 2:** Summary of studies reporting on the sexual impact of BV.

Study population	Sample size	Definition of (recurrent) BV	Study design	Major findings	Study ID
NP, symptomatic BV	139	Nugent OR Nugent score 3–6 and ≥ 3 Amsel criteria	Quantitative	84% and 43% of women experienced a mild-severe impact on their sex life respectively before treatment and after treatment.	Bradshaw et al. 2006 [11]
NP, symptomatic BV	404	Nugent score 3–10 and ≥ 3 Amsel criteria	Quantitative	88.6% of women experienced a mild-severe impact on their sexual satisfaction.	Bradshaw et al. 2013 [18]
African-American women with recurrent BV	20	≥ 2 proven episodes using Amsel criteria	Qualitative/ Quantitative	The relationship with their partner was affected in 95% of the women.	Payne et al. 2010 [27]
Women with recurrent BV	35	≥ 2 self-reported episodes per year	Qualitative	Sexual impact was the biggest impact of BV, in comparison with the emotional and social impact ^a^.	Bilardi et al. 2013 [7]
Women of heterosexual couples	252	Nugent	Quantitative	There was a decrease of 8,27 points on the SQoL scale when BV diagnosis and recent sexual intercourse concurred.	Mehta et al. 2018 [23]

**Legend.** NP, non-pregnant. SQoL, Sexual Quality of Life. ^a^ Author’s data did not allow us to present specific numbers here

Four studies found that BV symptoms had an impact on women’s sexual lives and sexual intimacy. In the prospective cohort study of Mehta and colleagues, the score of the Sexual Quality of Life Questionnaire decreased with 8,27 points on a scale of 100 when BV diagnosis (assessed with Nugent score) concurred with recent sexual activity, and this decrease was more likely to increase with age [23].

The qualitative study of Bilardi and colleagues reported that the degree of the impact on women’s sexual health was associated with the severity of the symptoms and the frequency of recurrences [7]. Interviews conducted in the qualitative studies, found that women were very self-conscious, embarrassed about having vaginal odor and feared that sexual partners may notice their symptoms, especially during oral sex [7, 27]. This resulted in not being able to relax and enjoy sex, negatively affected sexual self-esteem, sexual confidence and levels of intimacy with partners [7]. Women frequently associated their sexual attractiveness with a non-odorous vagina [7]. Most women with symptomatic BV demonstrated avoidance behavior, such as avoiding certain sexual positions and practices, particularly oral sex, and planned sexual activity after genital hygienic practices or abstained from sex altogether [7, 27]. A slight improvement of sexual enjoyment was observed one month after treatment with antibiotics [11]. Some women worried about infecting their partners when having sexual intercourse [7]. Interestingly, women did not think that their disease could have been transmitted from their partner and did not doubt their partner’s fidelity [7]. Bilardi and colleagues observed no differences in the impact of BV on sexual health between heterosexual women and women who have sex with women [7]. In general, women in relationships experienced greater support and encouragement compared to single women [7].

### Social health

Four studies reported on social health and BV and are summarized in Table 3 [7, 11, 26, 27]. Two studies included women suffering from recurrent episodes of BV [7, 27]. One study quantified the social impact with a social integration score based on the level of engagement in four activities: religious activities, participation in a community/voluntary group, social outings to meet friends and/or relatives, and hosting friends and/or relatives [26]. In two studies, a combination of questionnaires and interviews were used to obtain data, [11, 27]. Only one of the four studies primarily relied on interviews [7].

**Table 3:** Summary of studies reporting on the social impact of BV.

Study population	Sample size	Definition of (recurrent) BV	Study design	Major findings	Study ID
NP	2494	Nugent	Quantitative	OR low social integration – BV prevalence: 1.10 (95% CI 0.8–1.4), p = 0.74	Patel et al. 2006 [26]
NP, symptomatic BV	139	Nugent OR Nugent score 3–6 and ≥ 3 Amsel criteria	Quantitative	93% and 51% of women experienced a mild-severe impact on their everyday life respectively before treatment and after treatment.	Bradshaw et al. 2006 [11]
Women with recurrent BV	20	≥ 2 proven episodes using Amsel criteria	Qualitative/ quantitative	Social activities were affected in 80% of women and job was affected in 60% of women. 95% of the women felt uncomfortable around others.	Payne et al. 2010 [27]
Women with recurrent BV	35	≥ 2 self-reported episodes per year	Qualitative	Only a few women reported feeling self-conscious or uncomfortable at work^a^ when having BV whereas 4 out of 6 sex workers reported a substantial impact.	Bilardi et al. 2013 [7]

**Legend.** NP, non-pregnant; OR, Odds Ratio; CI, Confidence Interval. ^a^ Author’s data did not allow us to present specific numbers here

The proportion of women with BV affecting their social lives varied between the studies. In the cross-sectional study of Patel and colleagues, no statistically significant association was found between the social integration score and BV as assessed by the Nugent score [26]. Other studies reported that BV impacted social health and was associated with the severity of the symptoms and the frequency of recurrences [7, 27]. Bilardi and colleagues reported that only a minority suffered from social consequences of BV, [7]. whereas Payne and colleagues found more than half of the women reported a negative impact on work attendance, job performance and productivity, and relationships with coworkers [27]. The type of employment was shown to influence the impact of BV on social interactions on the work floor [7]. Women working in close contact with other people, such as teachers, health-care workers and especially sex workers, were more likely to suffer an impact on their work life [7, 16]. Shame and fear that others may detect their symptoms were important contributing factors [7, 27]. Avoidance behavior was common, ranging from avoiding side-by-side contact with colleagues to absenteeism. Women reported being reluctant to use public restrooms and tended to engage in frequent feminine hygienic practices, including douching [27]. Additionally, some women limited social interactions or avoided going out altogether [7, 27].

## Discussion

### Main findings

Overall, we found only few studies that investigated the association between BV and emotional, sexual and social health and most of these specifically addressed emotional health. There was a large heterogeneity between studies in study design, population and methodology. Four studies reported a statistically significant association between stress and BV, [20, 24, 25, 29] but hypothesized that stress is a risk factor for the development of BV. Because of the cross-sectional nature of these studies, that hypothesized that stress could be a risk factor for the development of BV, the reported associations could also point out the reverse hypothesis, namely BV causing more stress. This reverse causation is not unlikely and should be considered as well. In qualitative studies on BV and emotional health, many women reported being affected by the symptoms. The studies that investigated the association between BV and sexual health reported that, when symptoms were present, many women were less satisfied with their sexual life. Very few studies assessed the association between BV and women’s social lives, and this was reported to be less strong than the association with sexual health.

### Strengths and limitations

To our knowledge, this is the first scoping review summarizing the knowledge on the association between BV and emotional, sexual and social health. This review presents a detailed summary of the findings of the included studies, following the recommendations of the PRISMA-ScR statement. Yet several limitations need to be considered. First, following the large heterogeneity between studies, making comparisons was very difficult. A separate review of the qualitative respectively quantitative studies could have resulted in a more thorough analysis of the data. A thorough critical appraisal of the studies by using a quality assessment tool, has not been conducted. Second, because we limited our search strategy to three databases, additional studies could be identified in other databases. There may be additional search terms we did not use that could have given more hits. This study was further limited by the absence of search for grey literature in the search strategy.

### Further implications

The full burden of BV has been understudied, which makes it difficult to draw conclusions. At least two or three big quantitative studies performed in a sample of the general population about the extent of the association between BV and emotional, sexual, and social health, are required to assess the burden of BV. As a comparison, far more studies have researched the psychosocial burden of vulvovaginal candidiasis (VVC) [30–33] and vaginitis in general, [34, 35] including some large quantitative studies, although these diseases are less prevalent than BV. VVC has overlapping symptoms with BV (discharge being the most prominent), and VVC has been shown to clearly impact the quality of women’s lives [30–33]. Additionally, BV is frequently misdiagnosed as VVC hence mistreated by clinicians [17, 36]. The lack of knowledge on BV and its possible impact may reflect that it is still an enigmatic and misunderstood condition. It may also reflect the clinician’s focus on the pure physical presentation of a condition. However, the impact on quality of life can have further consequences. A decrease in sexual quality of life is associated with increased frustration and distress, anxiety, depression and relationship disruptions, [37] as well with decreased overall quality of life [38]. There is evidence that anxiety about sex and lacking enjoyment in sex is strongly associated with pain during intercourse, which may further downgrade sexual health [39]. Also, the possible impact of BV may have economic implications. One study reported that more than half of the women often missed days of work due to the shame of the heavy degree of discharge and/or odor. A recent review estimated the global annual cost for treatment of BV on 4.8 billion dollars, half of which was due to recurrent BV [4]. Absenteeism could be an extra cost for employers and society. Additionally, the health literacy of BV is also quite poor [17]. Many women had never heard of BV before their diagnosis [17] and wrongly assume the condition results from poor hygiene. This has been reported to lead to a wide range of intravaginal practices, such as wiping, cleansing, douching or the insertion of substances into the vagina, [16, 40] further exacerbating symptoms and associated negative feelings [36]. To improve the management of BV, it is therefore important that clinicians acknowledge the possible impact of symptomatic BV. There is clearly a need for better information from practitioners and/or other trusted sources. A review of the literature on the experiences and strategies of women to ameliorate symptoms could provide valuable information as well for therapeutic management.

## Conclusion

This review shows that symptomatic BV can be associated with diminished emotional, sexual and social health, but there is too little evidence to state the extent of this association nor to comment on the impact of BV on quality of life. Future large quantitative studies on the impact of BV may be of importance to boost research on therapeutic options and preventative measures, which will improve the overall management of BV.

## Electronic supplementary material

Below is the link to the electronic supplementary material.

Additional File 1: Preferred Reporting Items for Systematic reviews and Meta-Analyses extension for Scoping Reviews (PRISMA-ScR) Checklist

Additional File 2: PRISMA 2020 flow diagram for new systematic reviews which included searches of databases and registers only

Additional File 3: Search Strategy

Additional File 4: Table S1: Characteristics of included studies

Additional File 4: Table S3: Characteristics of excluded studies

Additional File 6: Table S3: Summary of studies reporting on the association stress and BV

## Data Availability

All data generated or analyzed during this study are included in this published article and its supplementary information files.

## List of abbreviations

BV: Bacterial Vaginosis
CI: Confidence Interval
NP: non-pregnant
OR: Odds Ratio
PRISMA-ScR: Preferred Reporting Items for Systematic Reviews and Meta-analyses extension for Scoping Reviews
SQoL: Sexual Quality of Life
STI: Sexually Transmitted Infections
VVC: vulvovaginal candidiasis
