# Supplementary material for: Scoping review of the association between bacterial vaginosis and emotional, sexual and social health
Source: BMC Womens Health. 2023 Apr 7;23:168. doi: 10.1186/s12905-023-02260-z (PMC10080849; doi:10.1186/s12905-023-02260-z)
Supplement: Supplementary file 2 — Additional File 2: PRISMA 2020 flow diagram for new systematic reviews which included searches of databases and registers only [file 12905_2023_2260_MOESM2_ESM.docx]

**Identification of studies via databases and registers**

Records removed *before screening*:

Duplicate records removed (n = 750)

Records marked as ineligible by automation tools (n = 0)

Records removed for other reasons (n = 0)

Records identified from*:

Databases (n = 1855)

Registers (n = 0)

**Identification**

Records screened

(n = 1105)

Records excluded**

(n = 1069)

Reports sought for retrieval

(n = 36)

Reports not retrieved

(n = 0)

**Screening**

Reports excluded:

Not reporting specifically about BV (n = 8)

No data on emotional, sexual or social burden of BV (n = 11)

Reporting on men’s experiences (n = 1)

Reports assessed for eligibility

(n = 36)

Studies included in review

(n = 16)

Reports of included studies

(n = 16)

**Included**

*Consider, if feasible to do so, reporting the number of records identified from each database or register searched (rather than the total number across all databases/registers).

**If automation tools were used, indicate how many records were excluded by a human and how many were excluded by automation tools.

From: Page MJ, McKenzie JE, Bossuyt PM, Boutron I, Hoffmann TC, Mulrow CD, et al. The PRISMA 2020 statement: an updated guideline for reporting systematic reviews. BMJ 2021;372:n71. doi: 10.1136/bmj.n71
