# Supplementary material for: Scoping review of the association between bacterial vaginosis and emotional, sexual and social health
Source: BMC Womens Health. 2023 Apr 7;23:168. doi: 10.1186/s12905-023-02260-z (PMC10080849; doi:10.1186/s12905-023-02260-z)
Supplement: Supplementary file 3 — Additional File 3: Search Strategy [file 12905_2023_2260_MOESM3_ESM.docx]

Search Strategy

| **Database** | **Search string** |
| --- | --- |
| Pubmed/MEDLINE (1946 – 14/11/2020) | ((bacterial vaginosis[All Fields]) OR (bacterial vaginosis[MeSH Terms])) AND ('burden' OR 'discomfort' OR (impact[All Fields]) OR (perceived stress scale[All Fields]) OR 'psychology' OR 'quality of life' OR (self-esteem[All Fields]) OR 'sexual life' OR 'shame' OR 'social life' OR 'stress') |
| Embase (1974 – 14/11/2020) | 'bacterial vaginosis’:ab,ti AND (burden OR discomfort OR impact OR 'perceived stress scale' OR psychology OR 'quality of life' OR 'self esteem' OR 'sexual life' OR shame OR 'social life' OR stress) |
| Web of Science (1964 – 14/11/2020) | TS = 'bacterial vaginosis' AND ALL = (burden OR discomfort OR impact OR 'perceived stress scale' OR psychology OR 'quality of life' OR 'self esteem' OR 'sexual life' OR shame OR 'social life' OR stress) |
