# Supplementary material for: Scoping review of the association between bacterial vaginosis and emotional, sexual and social health
Source: BMC Womens Health. 2023 Apr 7;23:168. doi: 10.1186/s12905-023-02260-z (PMC10080849; doi:10.1186/s12905-023-02260-z)
Supplement: Supplementary file 4 — Additional File 4: Table S1: Characteristics of included studies [file 12905_2023_2260_MOESM4_ESM.docx]

Table S1 - Characteristics of included studies

| **Author** | **Country** | **Study design** | **Study type** | **Total participants** | **Population** | **Definition of BV** | **Symptomatic BV** | **Correction for confounders** |
| --- | --- | --- | --- | --- | --- | --- | --- | --- |
| Anstey-Watkins et al. 2019 | UK | cross-sectional | qualitative | 33 | non-pregnant women | Self-report | 100% | N/A |
| Bilardi et al. 2013 | Australia | cross-sectional | qualitative | 35 | women suffering from recurrent BV | Self-report (2 or more episodes in 1 year) | 100% | N/A |
| Bilardi et al. 2016 | Australia | cross-sectional | qualitative | 35 | women suffering from recurrent BV | Self-report (2 or more episodes in 1 year) | 100% | N/A |
| Bradshaw et al. 2006 | Australia | prospective | quantitative | 139 | non-pregnant women | Nugent Gram stain score of ≥7 OR Nugent Gram stain score 3-6 and ≥3 Amsel criteria | 100% | yes |
| Bradshaw et al. 2013 | Australia | prospective | quantitative | 404 | non-pregnant women | Nugent Gram stain score 3-10 and ≥3 Amsel criteria | 100% | yes |
| Culhane et al. 2001 | USA | cross-sectional | quantitative | 454 | pregnant women | Nugent Gram stain score of ≥7 | Not defined | yes |
| Culhane et al. 2002 | USA | cross-sectional | quantitative | 2304 | pregnant women | Nugent Gram stain score of ≥7 | Not defined | yes |
| Harville et al. 2005 | USA | cross-sectional | quantitative | 411 | non-pregnant women (afro-american) | Nugent Gram stain score of ≥7 | Not defined | yes |
| Harville et al. 2007 | USA | prospective | quantitative | 897 | pregnant women | Nugent Gram stain score of ≥7 | Not defined | yes |
| Mehta et al. 2018 | Kenya | prospective | quantitative | 252 | women of heterosexual couples | Nugent Gram stain score of ≥7 | Not defined | yes |
| Nansel et al. 2006 | USA | prospective | quantitative | 3614 | non-pregnant women | Nugent Gram stain score of ≥7 | Not defined | yes |
| Nelson et al. 2008 | USA | cross-sectional | quantitative | 1916 | pregnant women | Nugent Gram stain score of ≥7 | 33,3% | yes |
| Patel et al. 2006 | India | cross-sectional | quantitative | 2494 | non-pregnant women | Nugent Gram stain score of ≥7 | 38,89% | yes |
| Payne et al. 2010 | USA | cross-sectional | quantitative/qualitative | 20 | women suffering from recurrent BV (afro-american) | Amsel criteria | 100% | N/A |
| Ruiz et al. 2001 | USA | prospective | quantitative | 78 | pregnant women | 2 out of 3 Amsel criteria (homogeneous discharge not included) | Not defined | no |
| Turpin et al. 2019 | USA | prospective | quantitative | 2439 | non-pregnant women | Nugent Gram stain score of ≥7 | Not defined | yes |
