# Supplementary material for: Scoping review of the association between bacterial vaginosis and emotional, sexual and social health
Source: BMC Womens Health. 2023 Apr 7;23:168. doi: 10.1186/s12905-023-02260-z (PMC10080849; doi:10.1186/s12905-023-02260-z)
Supplement: Supplementary file 5 — Additional File 4: Table S3: Characteristics of excluded studies [file 12905_2023_2260_MOESM5_ESM.docx]

Table S2 - Characteristics of excluded studies

| **Author** | **Title** | **Reason for exclusion** |
| --- | --- | --- |
| Bilardi et al. 2016 | Women's views and experiences of the triggers for onset of bacterial vaginosis and exacerbating factors associated with recurrence | No data on emotional, sexual or social burden of BV |
| Bilardi et al. 2017 | Women view key sexual behaviours as the trigger for the onset and recurrence of bacterial vaginosis | No data on emotional, sexual or social burden of BV |
| Boskey et al. 2004 | Acceptability of a self-sampling technique to collect vaginal smears for gram stain diagnosis of bacterial vaginosis | No data on emotional, sexual or social burden of BV |
| Bradshaw et al. 2014 | The influence of behaviors and relationships on the vaginal microbiota of women and their female partners: the WOW Health Study | No data on emotional, sexual or social burden of BV |
| Crann et al. 2018 | Vaginal health and hygiene practices and product use in Canada: a national cross-sectional survey | Not reporting specifically about BV |
| Guaschino et al. 2008 | SOPHY project: an observational study of vaginal pH, lifestyle and correct intimate hygiene in women of different ages and in different physiopathological conditions. | Not reporting specifically about BV |
| Guédou et al. 2013 | Behavioural and medical predictors of bacterial vaginosis recurrence among female sex workers: longitudinal analysis from a randomized controlled trial | No data on emotional, sexual or social burden of BV |
| Johnson et al. 2010 | Attitudes and experience of women to common vaginal infections | Not reporting specifically about BV |
| Karasz et al. 2003 | The vaginitis monologues: women's experiences of vaginal complaints in a primary care setting | Not reporting specifically about BV |
| Kenyon et al. 2018 | Association between bacterial vaginosis and partner concurrency: a longitudinal study | No data on emotional, sexual or social burden of BV |
| Klebanoff et al. 2004 | Vulvovaginal symptoms in women with bacterial vaginosis | No data on emotional, sexual or social burden of BV |
| Kostick et al. 2010 | Treatment Seeking, Vaginal Discharge and Psychosocial Distress Among Women in Urban Mumbai | Not reporting specifically about BV |
| Koumans et al. 2007 | The prevalence of bacterial vaginosis in the United States, 2001–2004: Associations with symptoms, sexual behaviors, and reproductive health. | No data on emotional, sexual or social burden of BV |
| Kurewa et al. 2010 | The burden and risk factors of Sexually Transmitted Infections and Reproductive Tract Infections among pregnant women in Zimbabwe | No data on emotional, sexual or social burden of BV |
| Marrazzo et al. 2006 | Women's satisfaction with an intravaginal Lactobacillus capsule for the treatment of bacterial vaginosis | No data on emotional, sexual or social burden of BV |
| Parsapure et al. 2016 | Impact of Health-Promoting Educational Intervention on Lifestyle (Nutrition Behaviors, Physical Activity and Mental Health) Related to Vaginal Health Among Reproductive-Aged Women With Vaginitis | Not reporting specifically about BV |
| Patel et al. 2005 | Why do women complain of vaginal discharge? A population survey of infectious and pyschosocial risk factors in a South Asian community | Not reporting specifically about BV |
| Vodstrcil et al. 2015 | Incident bacterial vaginosis (BV) in women who have sex with women is associated with behaviors that suggest sexual transmission of BV | No data on emotional, sexual or social burden of BV |
| Walraven et al. 2001 | The burden of reproductive-organ disease in rural women in The Gambia, West Africa | Not reporting specifically about BV |
| Wigan et al. 2020 | “It’s just an issue and you deal with it… you just deal with it, you move on and you do it together.”: Men’s experiences of bacterial vaginosis and the acceptability of male partner treatment | Reporting on men’s experiences |
| Yen et al. 2003 | Bacterial vaginosis in sexually experienced and non-sexually experienced young women entering the military. | No data on emotional, sexual or social burden of BV |
