# Supplementary material for: Scoping review of the association between bacterial vaginosis and emotional, sexual and social health
Source: BMC Womens Health. 2023 Apr 7;23:168. doi: 10.1186/s12905-023-02260-z (PMC10080849; doi:10.1186/s12905-023-02260-z)
Supplement: Supplementary file 6 — Additional File 6: Table S3: Summary of studies reporting on the association stress and BV [file 12905_2023_2260_MOESM6_ESM.docx]

**Supplementary Table S3.**

Summary of studies reporting on the association stress and BV.

| **Study population** | **Sample size** | **Definition of BV** | **Stress definition / measure** | **Relationship** | **Major findings** | **Study ID** |
| --- | --- | --- | --- | --- | --- | --- |
| P | 454 | Nugent | Q1 of PSS scores (14-item) | High chronic stress – BV prevalence | **aOR 2.2^£^ (1.1 – 4.2)** | Culhane et al. 2001^20^ |
|  |  |  | Q2 of PSS scores | Moderate-high chronic stress – BV prevalence | **aOR 2.3^£^ (1.2 – 4.3)** |  |
|  |  |  | Q3 of PSS scores | Low-moderate chronic stress – BV prevalence | aOR 1.3^£^ (0.7 - 2.4) |  |
|  |  |  | PSS | Mean stress score in BV+ vs BV- | **24.6 (BV) vs 22.2; p <.01** |  |
|  |  |  | PSS | Association between increasing chronic stress scores and increasing probability of BV | **Approximately linear** |  |
| P | 2304 | Nugent | Above median of PSS scores (14-item) | Stress – BV prevalence | aOR 1.2 (0.9 – 1.5) | Culhane et al. 2002^19^ |
| NP Afro-American | 411 | Nugent | GARS score of 14+ | Stress – BV prevalence | aRR 1.39 (0.95 – 2.05) | Harville et al. 2005^21^ |
| P | 897 | Nugent | Q1 of PSS scores (14-item) | High stress – BV prevalence | aOR 1.4^£^ (0.8 – 2.5) | Harville et al. 2007^22^ |
|  |  |  | Q2 of PSS scores | Medium-high stress – BV prevalence | aOR 1.0^£^ (0.6 – 1.8) |  |
|  |  |  | Q3 of PSS scores | Medium-low stress – BV prevalence | aOR 0.9^£^ (0.5 – 1.6) |  |
|  |  |  | Q1 of SAI scores | High stress – BV prevalence | aOR 1.3 (0.7–2.4) |  |
|  |  |  | Q2 of SAI scores | Medium-high stress – BV prevalence | aOR 0.7 (0.3–1.4) |  |
|  |  |  | Q3 of SAI scores | Medium-low stress – BV prevalence | aOR 1.3 (0.7–2.3) |  |
|  |  |  | Q1 of TAI scores | High stress – BV prevalence | aOR 1.4 (0.8 – 2.5) |  |
|  |  |  | Q2 of TAI scores | Medium-high stress – BV prevalence | aOR 1.0 (0.6 – 1.8) |  |
|  |  |  | Q3 of TAI scores | Medium-low stress – BV prevalence | aOR 0.9 (0.5 – 1.6) |  |
|  |  |  | Q1 of LES scores (negative life events) | High stress – BV prevalence | aOR 1.2 (0.7 – 2.1) |  |
|  |  |  | Q2 of LES scores (negative life events) | Medium-high stress - BV prevalence | aOR 0.8 (0.5 – 1.4) |  |
|  |  |  | Q3 of LES scores (negative life events) | Medium-low stress - BV prevalence | aOR 1.1 (0.7 – 1.7) |  |
|  |  |  | Q1 of LES scores (total life events) | High stress – BV prevalence | aOR 1.3 (0.7 – 2.4) |  |
|  |  |  | Q2 of LES scores (total life events) | Medium-high stress - BV prevalence | aOR 0.7 (0.4 – 1.4) |  |
|  |  |  | Q3 of LES scores (total life events) | Medium-low stress - BV prevalence | aOR 0.9 (0.5 – 1.7) |  |
|  |  |  | Q1 of JHCS scores | High stress – BV prevalence | aOR 1.8 (0.8 – 4.2) |  |
|  |  |  | Q2 of JHCS scores | Medium-high stress – BV prevalence | aOR 1.4 (0.6 – 3.3) |  |
|  |  |  | Q3 of JHCS scores | Medium-low stress – BV prevalence | aOR 1.2 (0.5 – 2.9) |  |
|  |  |  | Q1 of MOS-SS scores | High stress – BV prevalence | aOR 1.7 (0.8 – 3.4) |  |
|  |  |  | Q2 of MOS-SS scores | Medium-high stress – BV prevalence | aOR 1.1 (0.5 – 2.1) |  |
|  |  |  | Q3 of MOS-SS scores | Medium-low stress – BV prevalence | aOR 1.4 (0.7 – 2.6) |  |
|  |  |  | Q1 of cortisol measures | High cortisol – BV prevalence | aOR 1.3 (0.8 – 2.0) |  |
|  |  |  | Q2 of cortisol measures | Medium-high cortisol – BV prevalence | aOR 1.2 (0.8 – 1.9) |  |
|  |  |  | Q3 of cortisol measures | Medium-low cortisol – BV prevalence | aOR 1.5 (1.0 – 2.3) |  |
|  |  |  | Q1 of CRH measures | High CRH – BV prevalence | aOR 1.0 (0.6 – 1.6) |  |
|  |  |  | Q2 of CRH measures | Medium-high CRH – BV prevalence | aOR 1.1 (0.7 – 1.7) |  |
|  |  |  | Q3 of CRH measures | Medium-low CRH – BV prevalence | aOR 1.2 (0.8 – 1.8) |  |
| NP | 3614 | Nugent | PSS (10-item) | Stress (per PSS point increase) – BV prevalence | **aOR 1,10 (1,01 – 1,20)** | Nansel et al. 2006^24^ |
|  |  |  | PSS | Stress (per PSS point increase) – BV incidence | **aOR 1,29 (1,12 – 1,48)** |  |
| P | 1916 | Nugent | PSS (14-item) | Mean stress score in women with asymptomatic BV (cases) vs women with symptomatic BV (controls) | **aRR 0,78 (0,67 – 0,89)** | Nelson et al. 2008^25^ |
| P | 78 | Modified Amsel^#^ | PSS (10-item) | Stress level in BV vs no BV | No correlation found^@^ | Ruiz et al. 2001^28^ |
| NP | 2439 | Nugent | PSS (10-item) | Mean stress score in BV vs normal vaginal microbiome | **16.78 vs 17.46 (BV)** | Turpin et al. 2019^29^ |

**Legend Table S3**

aOR, adjusted odds ratio (documented with 95% confidence interval in between brackets); aRR, adjusted relative risk (documented with 95% confidence interval in between brackets); GARS, Global Assessment of Recent Stress, normally based on scoring seven areas of life from 0-9, but here alternatively calculated, giving a score of 1, 2 or 3 respectively for the intervals 0-3, 4-6 or 7-9. All scores were added up, with a minimum of 7 and a maximum of 21; JHCS, John Henryism Coping Style, used as instrument for stress measurement; LES, Life Event Questionnaire, used as instrument for stress measurement; MOS-SS, Medical Outcomes Study Social Support Survey, used as instrument for stress measurement; NP, non-pregnant; OR, odds ratio (documented with 95% confidence interval in between brackets); P, pregnant; PSS, Perceived Stress Scale, Q1-Q4, quartile 1 (highest quartile) to quartile 4 (lowest quartile, which is the reference quartile); RR, relative risk (documented with 95% confidence interval in between brackets); SAI, State Anxiety Inventory as measured by the Spielberger-State-Trait-Anxiety Inventory, used as instrument for stress measurement; TAI, Trait Anxiety Inventory as measured by the Spielberger-State-Trait-Anxiety Inventory, used as instrument for stress measurement; ^£^ compared to low stress (the reference group, the lowest quartile of the PSS scale); ^#^ BV defined when 2 of the following 3 criteria were met: (i) pH >4.5, (ii) >75% of the slide showing clue cells, (iii) a positive “whiff” test following the application of potassium hydroxide to the specimen; *^@^* No exact data or analysis were given in the manuscript; bold, statistically significant
